# Treatment of Bullous Pemphigoid with Avdoralimab: Multicenter, Randomized, Open-Labeled Phase 2 Study

**DOI:** 10.1016/j.xjidi.2024.100307

**Published:** 2024-08-23

**Authors:** Thierry Passeron, Eric Fontas, Thierry Boye, Marie-Aleth Richard, Emmanuel Delaporte, Olivier Dereure

**Affiliations:** 1 Department of Dermatology, Centre Hospitalier Universitaire de Nice, University Côte D’Azur, Nice, France; 2 INSERM U1065, C3M, University Côte D’Azur, Nice, France; 3 Department of Clinical Research and Innovation, Centre Hospitalier Universitaire de Nice, University Côte d'Azur, Nice, France; 4 Department of Dermatology, Sainte Anne Military Hospital, Toulon, France; 5 Department of Dermatology, University Hospital Timone, Assistance Publique Hôpitaux de Marseille, APHM, Marseille, France; 6 Department of Dermatology, North Hospital, Assistance Publique des Hôpitaux de Marseille, Marseille, France; 7 Department of Dermatology, Centre Hospitalier Universitaire de Montpellier, Montpellier, France

**Keywords:** Avdoralimab, Bullous pemphigoid, C5aR1

## Abstract

Rationale: Experimental data support the role for C5a–C5aR1 axis activation in bullous pemphigoid. We assessed the efficacy and safety of avdoralimab, a specific anti-C5aR1 mAb, for treating bullous pemphigoid. Methods: We conducted a phase 2 open-labeled randomized multicenter study. Patients with proven bullous pemphigoid were randomized (1:1) to receive superpotent topical steroids alone (group A) or with avdoralimab (group B). All patients received 0.05% clobetasol propionate cream until 15 days after the healing of lesions. Patients in group B additionally received 3 injections of avdoralimab every week for 12 weeks. The main criterion of evaluation was the proportion of patients with initial control of disease activity still in complete clinical remission at 3 months with no relapse during the study period. Results: Fifteen patients were randomized: 7 to group A and 8 to group B. Two patients in group A and in group B achieved control of disease activity at week 4. Only 1 patient was still in complete clinical remission at week 12 in group B, and none was in group A. No adverse event related to the treatment was reported. Conclusions: This proof-of-concept pilot study did not show preliminary signal of additional avdoralimab efficiency compared with superpotent topical steroids alone.

## Introduction

Bullous pemphigoid (BP), the most frequent skin autoimmune bullous disease, predominantly affects elderly patients aged >70 years with an estimated incidence of 21.7 new cases per million per year in France (Joly et al, 2012) and an overall poor prognosis compared with age- and gender-matched general population (Kridin et al, 2018; Li et al, 2013; Parker et al, 2008). Conventional therapies are mostly based on protracted applications of superpotent topical steroids (STS); high- or middle-dose systemic steroids; doxycycline; and immunosuppressive agents, including methotrexate. However, they are not systematically effective and may induce potentially severe adverse events (AEs) in a fragile elderly population (Amber et al, 2018). Besides deposition of autoantibodies against BP180 and/or BP230 antigens related to adaptive immune system activation, the innate immune system may also be involved in BP pathomechanisms mainly through local complement cascade activation. More specifically, the latter one can trigger the release of cytokines and chemokines, resulting in the recruitment and activation of inflammatory cells, including neutrophils and eosinophils (Edwards et al, 2019), leading to the occurrence of subepidermal blistering and inflammatory skin lesions. It has been established that prior neutrophil depletion protects wild-type mice against the development of experimental BP, whereas injection of C5a or IL-8 in *C5*-knockout mice led to a restored susceptibility for BP occurrence in this animal model (Liu et al, 1997). Moreover, the absence of C5aR1 expression on mast cells proved similarly protective against BP development, despite complement activation and the presence of neutrophils. These findings suggest that complement-dependent neutrophil recruitment and activation in BP might be related, at least partially, to C5aR1-induced mast cell degranulation (Chen et al, 2001; Heimbach et al, 2011). Further study confirmed the protection of *C5aR1*-knockout mice against BP occurrence, whereas *C5aR2*-knockout animals were more prone to disease development (Pigors et al, 2024). Eventually and as a proof of concept, prophylactic treatment of mice with a C5aR1 inhibitor PMX53 also reduced experimental disease development (Karsten et al, 2018). Altogether, these results support a significant role for a C5a–C5aR1 axis activation in BP pathomechanisms. Avdoralimab (IPH5401), a specific anti-C5aR1 mAb, has already been credited with a good safety profile in the treatment of patients with solid tumors, rheumatoid arthritis, or COVID-19 (Carvelli et al, 2022). The objective of this study was to investigate the additional clinical benefit of avdoralimab in association with STS compared with that of STS alone in patients with BP at 3 months.

## Results

**Figure 1:**
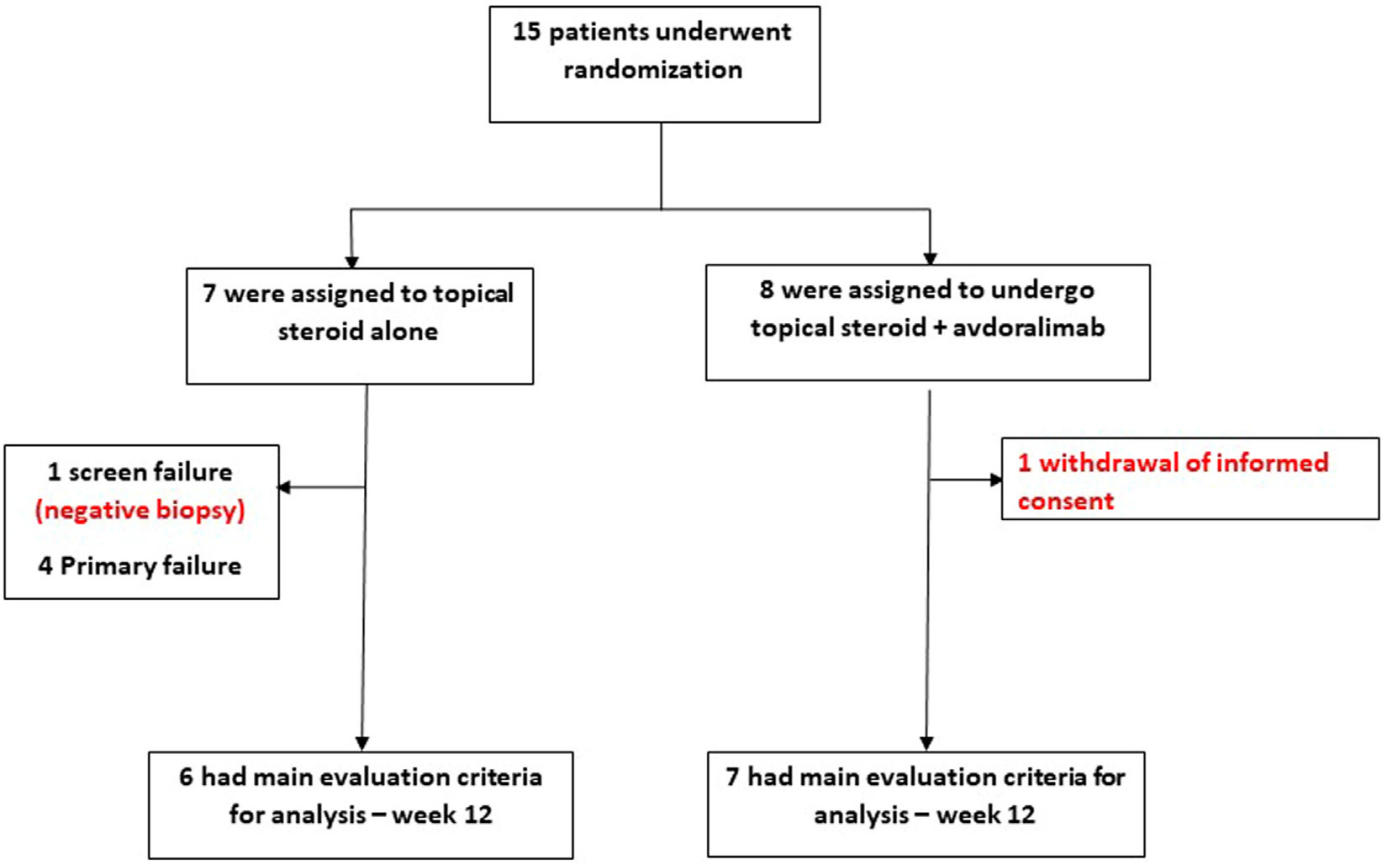
Flowchart of the study.

A total of 15 patients were randomized: 7 to group A and 8 to group B (Figure 1). The study was conducted between October 2020 and June 2022 and prematurely terminated owing to difficulties in recruitment while no signal of additional clinical improvement was observed in the experimental group compared with the group treated with STS alone. The mean age was 79 years in group A and 82 years in group B. The 2 groups were overall similar except for the initial pruritus score that was higher in group A. The population characteristics are described in Table 1.

**Table 1:** Characteristic of the Population

Characteristic	n	Topical Steroid Alone (n = 6)	n	Topical Steroid + Avdoralumab (n = 8)
		%		%
Demographics				
Age (y), mean (±SD)	6	79.3 (3.9)	8	82.1 (10.5)
Sex				
Male	2	33.3	5	62.5
Female	4	66.7	3	37.5
Medical history				
Disease duration (mo), median (range)	6	0.23 (0.03–12.3)	8	2.5 (0.07–32.6)
High blood pressure	3	50.0	7	87.5
Type II diabetes	5	83.3	4	50.0
Peripheral vascular disease	0	0.0	1	12.5
Myocardial infarction	1	83.3	0	0.0
Bullous pemphigoid activity				
Number of bullous lesions at inclusion, median (range)	6	30.0 (9–80)	8	17.0 (0.0–90.0)
VAS, median (range)	6	9.0 (4.0–10.0)	7	5.0 (2.0–8.0)
DLQI, median (range)	6	11.5 (3.0–15.0)	8	8.5 (2.0–26.0)
BP180 antibody levels, median (range)	6	34,554 (2000–211,935)	7	87,634 (2000–264,579)
BP230 antibody levels, median (range)	6	1610 (486–4179)	7	61,132 (1000–44,069)

Abbreviations: DLQI, Dermatology Life Quality Index; VAS, Visual Analog Scale.

VAS for pruritus is from 0 (none) to 10 (maximum).

Two patients in group A and 2 patients in group B achieved initial control of disease activity at week 4. Only 1 patient was still in complete clinical remission at week 12 in group B, and none was in group A. The evolution of the number of bullous lesions, presence of inflammatory lesions, pruritus, and Dermatology Life Quality Index are presented in Table 2. All these parameters initially improved but relapsed when steroids were stopped in both groups. The median (range) of eosinophil counts for avdoralimab + topical steroids and topical steroids alone were respectively 1250 (500–3600) cells/ml and 1000 (600–2700) cells/ml at baseline, 350 (0–3300) cells/ml and 1350 (100–2600) cells/ml at week 4, 600 (300–2000) cells/ml and 200 (100–300) cells/ml at week 8, and 400 (0–500) cells/ml and 250 (100–400) cells/ml at week 12. The serum levels of BP180 and BP230 remained high in both groups at all time points (Table 3). No AE was reported in group A, whereas 2 patients in the experimental arm experienced serious AEs (1 with hyperalgia from post bullous erosive lesions and 1 with multiple hemorrhagic strokes), but none of them was considered related to the treatment. No local reactions were observed at the injection sites.

**Table 2:** Evolution of Bullous Lesions, Inflammatory Lesions, Pruritus, and DLQI Overtime

Symptoms and Quality of Life	Treatment Arm	Week 0	Week 4	Week 8	Week 12	Week 16
Bullous lesions	Avdoralumab + steroids—plaques, median (range)	17.0 (0.0–90.0)	0.0 (0.0–5.0)	1.0 (0.0–2.0)	9.5 (0.0–19.0)	6.0 (0.0–20.0)
	Steroids alone—plaques, median (range)	30.0 (9.0–80.0)	0.0 (0.0–0.0)	0.0 (0.0–0.0)	0.0 (0.0–0.0)	26.0 (0.0–82.0)
Presence of inflammatory lesions	Avdoralumab + steroids—plaques, n (%)	7 (87.5)	1 (16.7)	1 (50.0)	0 (0.0)	3 (50.0)
	Steroids alone—plaques, n (%)	5 (83.3)	0.0 (0.0)	0.0 (0.0)	0.0 (0.0)	3 (50.0)
Pruritus on VAS	Avdoralumab + steroids—VAS, median (range)	5.0 (2.0–8.0)	1.0 (0.0–4.0)	1.5 (0.0–3.0)	5.5 (2.0–9.0)	5.0 (0.0–9.0)
	Steroids alone—VAS, median (range)	9.0 (4.0–10.0)	0.0 (0.0–0.0)	0.0 (0.0–0.0)	1.5 (0.0–3.0)	6.0 (6.0–7.0)
DLQI	Avdoralumab + steroids—DLQI, median (range)	8.5 (2.0–26.0)	3.0 (0.0–15.0)	0.5 (0.0–1.0)	6.0 (5.0–7.0)	7.0 (2.0–18.0)
	Steroids alone—DLQI, median (range)	11.5 (3.0–15.0)	0.5 (0.0–1.0)	0.5 (0.0–1.0)	1.0 (1.0–1.0)	8.0 (6.0–10.0)

Abbreviations: DLQI, Dermatology Life Quality Index; VAS, Visual Analog Scale.

**Table 3:** Evolution of Anti-BP180 and -BP230 Antibodies

Treatment Arm and Type of Antibodies	Week 0	Week 4	Week 8	Week 12
Avdoralumab + steroids—BP180 antibodies, median (range)	87,634 (2000–264,579)	19,846 (2000–71,257)	17,324 (6330–28,318)	17,752 (2000–162,659)
Steroids alone—BP180 antibodies, median (range)	34,554 (2000–211,935)	47,239 (23,137–71,341)	28,694 (15,231–42,157)	48,612 (2000–208,401)
Avdoralumab + steroids—BP230 antibodies, median (range)	4864 (1000–103,012)	17,934 (1000–44,069)	14,326 (2715–25,936)	11,225 (1000–44,622)
Steroids alone—BP230 antibodies, median (range)	1610 (487–4179)	2497 (1000–3994)	2713 (1000–4426)	7364 (994–28,145)

## Discussion

Despite a strong experimental rationale for using an anti-C5aR1 antibody in BP treatment, this proof-of-concept pilot study did not show preliminary signal of additional avdoralimab efficiency compared with STS alone. Limitations of this study are the number of patients finally included (lower than the initial calculated sample size) and the use of topical steroids that could have masked the effect of avdoralimab. To limit this bias, we limited the duration of STS use. No strong signal of additional efficacy was observed in the avdoralimab-treated group regarding both initial control of disease activity or maintenance because only 1 patient was in complete clinical remission at week 12 compared with none in STS alone.

Several reasons could explain the absence of efficacy of avdoralimab in treating BP. Most of the data reporting the role of C5aR1 in BP have been obtained from mouse models. It might be hypothesized that this pathway is less relevant in humans or that experimental BP uses pathways different from those in the spontaneous disease. Differences between murine and human innate immune system may be the cause for differences in neutrophils involvement. The region targeted by the autoantibodies may also contribute to this absence of efficacy in human. An alternative explanation might also be that targeting the complement pathway is ineffective when the disease has already been initiated, leading to active lesions occurrence. However, positive results have been recently reported with nomacopan, a leukotriene B4 and complement C5 inhibitor (Sadik et al, 2022), with 7 of 9 patients showing response, including 3 experiencing >80% improvement at week 6 in a phase 2a nonrandomized controlled trial, but it must be pointed out that lesional applications of mometasone furoate was allowed for the first 21 days. Although our results do not support the use of avdoralimab in treating BP and question the rationale of targeting the complement pathway in this autoimmune bullous disease, placebo-controlled studies seem warranted to assess the interest of targeting the complement in BP.

## Materials and Methods

### Study design and participants

This was a phase 2, multicentric, randomized, open-labeled, parallel-group study performed in 5 clinical sites in France.

Male and female patients aged >18 years with histologically and immunologically proven BP (direct immunofluorescence and/or ELISA positivity for BP180 and/or 230) were included. Noninclusion criteria included contraindication to STS, use of systemic steroid, any immunosuppressive drugs, doxycycline or minocycline, intravenous Igs within 4 weeks prior to inclusion, and use of rituximab or dupilumab in the past 12 weeks.

This study was conducted in accordance with the International Conference for Harmonisation guidelines, applicable regulations, and the Declaration of Helsinki. The study was approved by national institutional review board (2020-002912-34) and was registered on ClinicalTrial.gov (NCT04563923). All patients provided written informed consent before screening.

### Procedures

After written informed consent was signed, patients were randomized (1:1) to receive STS alone (group A) or STS with avdoralimab (group B). All patients received 0.05% clobetasol propionate cream (2 tubes of 10 g/day for patients <45 kg body weight and 3 tubes for patients of 45 kg and above) on a daily basis until 15 days after the healing of the last bullous lesion. Patients in group B additionally received 3 subcutaneous injections of avdoralimab (450 mg) every week for 12 weeks. The dose and schedule of 450 mg avdoralimab subcutaneous Q1W as monotherapy has been selected on the basis of pharmacokinetics/pharmacodynamics and safety data from avdoralimab phase I studies (NN8210-3926 and NN8210-3927).

### Outcomes

The number of bullous and inflammatory lesions along with pruritus assessed on a Visual Analog Scale were evaluated on a daily basis using a study diary. Clinical and biological assessments were performed at baseline, week 4, week 8, week 12, and week 16. At each visit, the number of new bullous and inflammatory lesions were noted. The pruritus was evaluated on a Visual Analog Scale from 0 (no pruritus at all) to 10 (the worst pruritus imaginable for the patient). The main criterion of evaluation was the proportion of patients achieving initial control of disease activity and still in complete clinical remission at 3 months with no relapse during the study period. The initial control of disease activity was defined as the absence of new bullous and skin inflammatory lesions and of pruritus (Visual Analog Scale for pruritus <3) for at least 2 weeks.

Safety was evaluated by the number and proportion of patients experiencing AEs. Vital signs were measured, laboratory tests were performed, and physical examinations were completed throughout the study. The following AEs were assessed: treatment-emergent AEs (TEAEs), serious AEs, TEAEs leading to death, TEAEs considered to be related to study drug, TEAEs leading to discontinuation of study drug, and any severe TEAEs (grade 3 or above according to National Cancer Institute Common Terminology Criteria for Adverse Events, version 5).

### Statistical analysis

Thirty-six evaluable patients were planned to obtain a 1-sided 95% confidence interval for a difference in proportions with lower bound at most far 0.19 units from the observed proportion. This sample size was calculated on the basis of the hypothesis of a 3-month disease control proportion of 0.05 and 0.25 in the control and experimental arms, respectively.

Continuous variables are expressed as means (±SD) or median (range) as appropriate and as absolute numbers and relative frequencies for categorical ones. This descriptive analysis was performed using SAS Enterprise Guide software, version 7.1 (Copyright 2017 by SAS Institute, Cary, NC).

## Ethics Statement

The study was approved by national institutional review board (2020-002912-34). A written informed consent was obtained for all the patients.

## Data Availability Statement

Anonymized data are available upon request for scientific purposes to EF (fontas.e@chu-nice.fr).

## ORCIDs

Thierry Passeron: http://orcid.org/0000-0002-0797-6570

Eric Fontas: http://orcid.org/0000-0002-1849-9626

Thierry Boyé: http://orcid.org/0009-0002-5855-1537

Marie-Aleth Richard: http://orcid.org/0000-0002-0870-9132

Emmanuel Delaporte: http://orcid.org/0009-0000-7057-0998

Olivier Dereure: http://orcid.org/0000-0001-8736-1922

## Conflict of Interest

TP has received grants and/or honoraria from AbbVie, ACM Pharma, Amgen, Almirall, Boehringer Ingelheim, Bristol-Myers Squibb, Calypso, Celgene, Galderma, Genzyme/Sanofi, GlaxoSmithKline, Incyte, Janssen, LEO Pharma, Eli Lilly, Novartis, Pfizer, Roivant, Sun Pharmaceuticals, UCB, and Vyne therapeutics. TP is the cofounder of YUKIN Therapeutics and has patent rights on the use of CXCR3B, WNT agonists, and GSK3b in the treatment of vitiligo. TB has received grants and/or honoraria from AbbVie, Amgen, Almirall, Eli Lilly, Janssen, Leo Pharma, Novartis, Pfizer, and UCB Pharma. M-AR has received grants and/or honoraria from AbbVie, ACM Pharma, Amgen, Almirall, Boehringer Ingelheim, Bristol-Myers Squibb, Genzyme/Sanofi, Incyte, Janssen, LEO Pharma, Eli Lilly, Novartis, Pfizer, and UCB. ED has received grants and/or honoraria from Abbvie, Amgen, Almirall, Janssen, Leo-Pharma, and Novartis. The remaining authors state no conflict of interest.
